# Migrating Anatidae as Sources of Environmental Contamination with Zoonotic *Giardia*, *Cryptosporidium*, *Cyclospora* and Microsporidia

**DOI:** 10.3390/pathogens12030487

**Published:** 2023-03-20

**Authors:** Piotr Solarczyk, Agnieszka Wojtkowiak-Giera, Mike Heddergott

**Affiliations:** 1 Department of Biology and Medical Parasitology, Poznan University of Medical Sciences, 10 Fredry Street, 61-701 Poznan, Poland; 2 Department of Zoology, Musée National d’Historire Naturelle, 25, Rue Münster, 2160 Luxembourg, Luxembourg

**Keywords:** *Giardia*, *Cryptosporidium*, *Cyclospora*, microsporidia, public health, epidemiology, wild birds, waterfowl

## Abstract

*Giardia*, *Cryptosporidium*, *Cyclospora*, and microsporidia are gastrointestinal pathogens that can cause various disease symptoms in both animals and humans. Numerous studies worldwide have confirmed the presence of these eukaryotic pathogens in nesting and migrating wild geese, ducks, and swans. Migration spreads these zoonotic enteric pathogens to distant locations, which could have public health implications. Soils and water bodies (lakes, ponds, rivers and wetlands) in urban and suburban areas have been shown to be vulnerable to contamination by waterfowl droppings. This review addresses the epidemiology of these enteric pathogens in wild migratory bird species (Anatidae) and some consequences of their spread in the environment. To date, both zoonotic pathogens and genotypes restricted to avian hosts have been found in faecal samples from 21 anatid species worldwide. One of the routes of infection for these zoonotic gastrointestinal micropathogens is the indirect route. For example, shared water bodies (e.g., for drinking or recreational purposes) previously contaminated by birds during the migratory season may facilitate infections of humans through water. However, it is unclear how much wild waterfowl contribute to the transmission of giardiasis, cryptosporidiosis, cyclosporosis, and microsporidiosis in many regions through contaminated environmental sources. Comprehensive epidemiological surveillance based on molecular data on gastrointestinal pathogens is crucial to take measures to control infections in the future.

## 1. Introduction

There are over 1400 described human pathogens, of which about 62% are classified as zoonotic [1]. In addition to bacterial, viral, and prion pathogens, a significant proportion of all zoonotic infections in humans are caused by unicellular, eukaryotic pathogens belonging to fungi and parasites [2]. Worldwide, 335 emerging infectious diseases have been described, 36 of which were caused by enteric protozoa (EP) including various species and genotypes of *Giardia duodenalis*, *Cryptosporidium* spp., and *Cyclospora* sp. as well as microsporidia [3]. Most EP and microsporidia surveillance studies involve domestic and farm animals as well as wildlife including migratory birds [4,5]. Wild and highly mobile migratory birds may play a role as a potential source of infection with public health implications. However, there is still no “gold standard” for the molecular study of such enteric parasites, so there are relatively few well-documented studies to date that have detected potentially zoonotic isolates of *G. duodenalis*, *Cryptosporidium* spp., and *Cyclospora* sp. as well as microsporidia in wild waterfowl. In addition, the seasonal and diurnal mobility of waterbirds makes it difficult to access adequate numbers of samples, forcing researchers to collect bird droppings from the ground rather than directly from captured birds. In addition, migratory birds congregate in mixed flocks during migration, making it impossible to determine which bird species is responsible for contamination of the soil or water with zoonotic pathogens [6,7,8,9]. The aim of this review is to summarise the role of wild migratory Anatidae waterfowl (ducks, geese and swans) in the spread of some enteric pathogens in humans, based on the molecular characterisation of *Giardia*, *Cryptosporidium*, *Cyclospora*, and microsporidia.

The EP and microsporidia studied have been detected in migratory waterfowl (Anatidae) as well as in humans [5]. These unicellular pathogens share common epidemiological characteristics. They are the most common agents of diarrhoeal disease in humans and animals worldwide after viral infections [10] and pose a serious threat to public health as the number of waterborne outbreaks in industrialised countries has increased over the last decade [10,11]. Their (oo)cysts/spores are resistant to environmental factors for months and are spread relatively easily via the faecal–oral route [12,13]. They are capable of causing waterborne outbreaks as they remain infectious for a long time, especially in association with aquatic habitats (e.g., ponds, lakes, and wetlands) [14,15]. They are capable of causing zoonotic infections, have been found in all species of vertebrates including geese, ducks, swans, and humans [8,16,17,18], with the exception of *C. cayetanensis*, and can cause significant economic losses in livestock that share fields and watersheds with migratory birds of the Anatidae [6,19,20]. They belong to the group of “emerging and re-emerging pathogens” and, with the exception of *C. parvum* and *G. duodenalis*, are not routinely diagnosed in animals and human patients [21,22]. Finally, their heterogeneity may lead to conflicting epidemiological conclusions about their zoonotic potency (e.g., different loci analysed instead of uniform molecular markers from different hosts, or one marker compared to multi-locus sequence typing) [4,17,23].

## 2. Materials and Methods

The review was supported by data received from scientific articles published in the PubMed and Science Direct database between 1998 and 2023, found in combination with the following keywords: ‘gastrointestinal agents’, ‘enteric protozoa’, ‘public health’, ‘epidemiology’ and ‘wild birds waterfowl’.

## 3. Results and Discussion

### 3.1. Bird Species Associated with Migration and Environment Degradation

The seasonal migration of waterfowl is one of the most spectacular natural phenomena. In total, an estimated five billion migratory birds migrate every autumn, representing more than 300 species worldwide [24]. Birds of the order Anseriformes (family Anatidae) are large migratory birds with long migration routes, crossing cities, parks, forests, farmland, reservoirs, and lakes during their autumn–winter migration [25]. In terms of human infections, bird migration provides a mechanism for the establishment of new and endemic disease foci at great distances from previous infection sites [24]. In addition, due to gradual climate change and negative human impacts, new areas and resting places are being explored by waterbirds [26]. Therefore, this review is limited to a group of waterbirds from the family Anatidae, as they can form large flocks during the migratory season [27] and often graze and defecate in both water and fields in urban or suburban areas [6]. In many regions where they have unrestricted access to the waters they share with humans, they are protected by environmental laws [28], are an essential component of the natural aquatic environment, and are necessary for the proper functioning of aquatic ecosystems [29].

Members of the family Anatidae can act as long-distance vectors for a wide range of different pathogens [30,31]. Free-ranging ducks, geese, and swans not only harbour human-infectious enteropathogens in their faeces, but can also be a source of infection for farm animals living in the same area [32]. The presence of waterbirds in spring water reservoirs has been linked to the declining quality of these waters [33]. The appearance of new populations and residence in flocks can rapidly increase the incidence of zoonotic *Giardia*, *Cryptosporidia*, *Cyclospora*, or microspodidia due to the eutrophication of wetlands [33,34,35,36,37]. As a rule, anseriform birds travel in large flocks, so their presence during migration affects the quality of the environment on a local scale [37]. Geese, ducks, and swans grouped in flocks tend to exploit heavily agricultural habitats through their scavenging, but no bioeconomic or risk assessment models have comprehensively integrated this aspect yet [38]. Furthermore, geese travelling in large flocks provide the opportunity and capacity to leave larger amounts of faeces in the habitats they use [16,36,37]. Each goose in the flock leaves 0.9 to 1.8 kg of faeces per day, with each faeces containing 25 times as many faecal bacteria as human waste [6,38]. Thus, 30 geese on a landscape plot (averaging 1.3 kg per day per goose) can leave behind an equal amount of 40 kg of faeces per day. The total weight of faeces collected after a single visit by a flock of geese was 12.6 kg (about 733 droppings) [33].

The model of pathogen deposition by waterfowl takes into account the number of faecal pellets deposited on a 1 m × 100 m section of shore during a single visit by an average flock [34]. Using this model for *Cryptosporidium* and *Giardia*, a single visit by an average flock of waterbirds may yield approximately 9.3 × 10^6^ g^−1^ of infective oocysts and 1.0 × 10^7^ g^−1^ cysts, respectively [33]. Unfortunately, there is no information on how many birds are accumulated in an average flock. Microsporidian spores (*E. hellem*) have also been found to be more abundant in avian faecal material than *Cryptosporidium*, reaching 9.1 × 10^8^ g^−1^ [39]. However, this model also needs to be verified for *C. cayetanensis*, zoonotic *E. intestinalis*, *E. cuniculi*, and *E. bieneusi* through field research. Overall, the circumstances under which anthroponotic or reverse zoonotic transmission of EP and microsporidia lead to zoonotic transmission are poorly understood [36].

### 3.2. Giardia

*Giardia duodenalis* (syns. *G. intestinalis*, *G. lamblia*) is one of the most common enteric protozoa in humans, infecting approximately 200 to 280 million people annually [40]. *Giardia duodenalis* exhibits considerable genetic diversity, with eight assemblages (A–H) reported. These assemblages differ in their host specificity, with mainly *G. duodenalis* assemblage A (AI and AII) and assemblage B (BIII and BIV sub-assemblages) causing human giardiasis [41,42]. Giardiasis can be asymptomatic or symptomatic and cause diarrhoea in the host. According to the literature, the likelihood of *Giardia* being spread in the environment by wild waterfowl during the seasonal migration period is high, but there is little information on the presence of zoonotic *Giardia* genotypes in wild migratory birds.

To date, *Giardia* surveys have been conducted in waterbird populations in four countries—USA, Poland, Spain and Iran—which included 12 species of Anatidae. Forty-five positive samples were found from 496 specimens in all countries. Four molecular markers (heat shock protein, giardin, 18S rRNA, and ITS1-5-8S-ITS2) were used for genotyping *Giardia* from migratory bird samples (Table 1). Zoonotic assemblages A and B of *G. duodenalis* have been detected in mallards (*Anas platyrhynchos*) in Spain, suggesting that this species is a vector of these pathogens infectious to humans [43]. Due to their aquatic lifestyle, ducks can be infected by water previously contaminated with the faeces of human or animal origin. It should be noted that the contamination of water bodies by human and other animal faecal material has been identified in this region, so the potential pathogen route for infections in wildlife could be from zooanthroponotic sources [43].

Although mallard, greylag goose (*Anser anser*), mute swan (*Cygnus olor*), and common merganser (*Mergus merganser*) have been implicated in the water-associated transmission of *G. duodenalis* in Poland, further molecular studies are needed on whether these spread zoonotic *Giardia* [45]. Despite the use of three molecular markers, there is also no evidence of *G. duodenalis* assemblages that could postulate clear epidemiological consequences in terms of public health in nine positive waterbird species from the USA [44,47] (Table 1). An epidemiological study of *Giardia* in greylag geese and tufted ducks (*Aythaya fuligula*) from Iran was based only on microscopic data [46]. Considering that no outbreak of giardiasis associated with faecal contamination by waterfowl has yet to be documented, further studies should be conducted to obtain more reliable information on *G. duodenalis* assemblages, cross-infections, and pathogenicity by these migratory avian hosts.

### 3.3. Cryptosporidium

*Cryptosporidium* is an important enteric protozoan of public health concern, spread mainly through water [54]. Although the global prevalence is 4.3% in developed countries and 10.4% in developing countries, the actual number of cases is still underestimated [57]. There are 44 valid *Cryptosporidium* species and >120 genotypes, which include both zoonotic and species-specific types [58,59]. Although *C. meleagridis*, *C. canis*, *C. felis*, *C. ubiquitum*, and 15 other *Cryptosporidium* genotypes are known to infect humans, most human infections are caused by *C. hominis* and *C. parvum* [59]. Nowadays, *Cryptosporidium* infections have been detected in 19 species of wild Anatidae waterfowl in 10 countries (Table 1). In total, there were 213 *Cryptosporidium*-positive samples out of 2350 samples collected from wild Anatidae birds worldwide (Table 1). There were three zoonotic *Cryptosporidium* species detected: *C. parvum* in most waterbird species such as Canada goose (*Branta canadensis*), mallard, taiga goose (*Anser fabalis*), mute swan, lesser merganser, whooper swan (*Cygnus cygnus*), and greylag goose; *C. hominis* and *C. meleagridis* in one Canada goose and one greylag goose species each [19,32,43,49,54]. Two human pathogenic genotypes of *C. meleagridis* (IIIgA22G3R1) and *C. parvum* (*C. parvum* genotype 2) were also found in greylag geese and Canada geese, respectively [33,51] (Table 1). Other wild Anatidae also carried two potentially zoonotic *Cryptosporidium* species: ruddy shelduck (*Tadorna ferruginea*) and mallard were infected with *C. baliyei*, while *C. andersoni* was detected in whooper swan and greylag goose [32,42,55,56] (Table 1). In addition, four avian subtypes were detected: *Cryptosporidium* goose genotype I and II in Canada goose and Whooper swan; avian genotype III (*C. proventriculi*) was detected in “migratory ducks” and *Cryptosporidium* duck genotype was found in Canada geese [32,53,54,55,56] (Table 1).

Although the 18S rRNA gene fragment (SSU-rDNA) has been most commonly used to determine species and genotypes, four other molecular markers such as the actin gene, the heat shock protein gene (HSP-70), the 60-kDa glycoprotein gene (*gp60*), and the thrombospondin-related adhesion protein (TRAP C2) have been successfully used for identifying *Cryptosporidium* species and genotypes in wild anatids (Table 1). The data suggest that wild waterbirds may be carriers of more than one potentially zoonotic *Cryptosporidium* species or genotype at a time [53].

### 3.4. Cyclospora

The genus *Cyclospora* includes 21 species of intestinal apicomplexan parasites of vertebrates and invertebrates, of which only *C. cayetanensis* can infect humans [60]. *C. cayetanensis* continues to raise questions about host specificity, infective dose to different hosts, aspects of sporulation, and external persistence of the parasite’s oocysts in the environment. Most observations are based on environmentally derived stages, as there are no animal models or in vitro culture systems to facilitate the study of *C. cayetanensis*, and all attempts to experimentally infect specific laboratory animals have been unsuccessful [61]. Therefore, little is known about the possible role of migratory waterbirds as potential *C. cayetanensis* reservoirs.

Microscopic and molecular methods have been used to describe *C. cayetanensis* in wild waterfowl (Anseriformes) in Poland. In the study, only samples from wild taiga geese and mute swans were positive (Table 1). The positive isolates obtained were confirmed by nested PCR with a fragment of small subunit ribosomal RNA. The results showed that these two waterbird species were infected with *C. cayetanensis* [62]. In addition to the similarities presented based on the morphology and morphometric measurements with human oocysts and molecular confirmation, further molecular studies are needed to evaluate geese and swans as a source of human cyclosporiasis [63,64]. The major drawback of the primers used in the study is the ability to cross-amplify other *Eimeria* DNA, which is non-specific to humans [64]. In addition, isolated oocysts of *C. cayetanensis* were found in the faeces of waterfowl in the study, suggesting that geese and swans were the mechanical vectors of the parasite. It is postulated that *C. cayetanensis* is a host-specific pathogen for humans that is rarely found in animals worldwide [63,64].

### 3.5. Microsporidia

Microsporidia are obligate and intracellular pathogens of invertebrates and vertebrates with over 1700 species described in insects, fish, crustaceans, mammals, and birds [65]. Human microsporidiosis can be caused by 17 opportunistic species from the *Encephalitozoon* spp. group, but mainly *E. cuniculi*, *E. intestinalis*, and *E. hellem* are found in infected individuals [65]. The clinical manifestations of microsporidiosis range from gastrointestinal disorders including enteritis to diffuse systemic infections without specific manifestations in immunocompetent and immunosuppressed individuals [65,66]. Although clinically relevant, the most important species with the widest host range is *Enterocytozoon bieneusi*. Other *Encephalitozoon* and *Enterocytozoon* species from the Microsporidia phylum are also important emerging pathogens [50].

A total of 559 faecal samples from four species of the family Anatidae were obtained in two countries, of which 47 were microsporidia-positive (Table 1). The DNA of *E. bieneusi* was found in whooper swans, while the spores of *E. hellem* were detected in mallards, greylag geese, and mute swans in China and Poland, respectively [16,39] (Table 1). The results suggest that such a large amount of zoonotic microsporidia in bird feed represents a natural infection rather than a mechanical transmission of the spores ingested by birds from the environment [39]. Based on the sequence and phylogenetic analyses of the internal transcribed spacer (ITS) of *E. bieneusi*, 11 genotypes including seven zoonotic ones (Peru6, EbpA, EbpC, Henan-III, CSW3, Henan-IV, and CSW1) were detected in the whooper swan [16]. Thus, in the context of public health, migratory swans may play an important role in the transmission of infectious *E. bieneusi* via water. The possibility of faecal contamination of field areas, parks, and water bodies by swans during migration is relatively high and may be the most direct link to human health risks in cities and sub-urban environments. Other wild waterfowl not belonging to the Anatidae family also spread potentially zoonotic microsporidium genotypes of *E. cuniculi* in Slovakia [35].

## 4. Conclusions and Future Perspectives

Although the direct transmission of *Giardia*, *Cryptosporidia*, *C. cayetanensis*, and microsporidia to humans by geese, ducks, and swans has not yet been demonstrated, and the importance of determining the various aspects of giardiasis, cryptosporidiosis, cyclosporosis, and microsporidiosis as a zoonosis originating from free-ranging waterfowl is undeniable. The main exposure to these micropathogens is via the indirect route (i.e., contaminated water eaten by waterfowl). Lakes, ponds, and lagoons as their habitat in suburban areas or urban public parks are the most direct route for human health exposure. This indirect route of exposure therefore makes it difficult to identify the source of these micropathogens from waterfowl. However, the actual link between (oo)cysts and spores of *Cyclospora*, *Cryptosporidium*, *Giardia*, and Microsporidia deposited by wild migratory birds and their concentrations in open waters is underestimated. More targeted research is needed to better assess the actual risk of the contamination of fields and waters with zoonotic micropathogens by wild migratory birds during migration. Management strategies to improve water quality need to take into account the potential contamination by migratory waterbirds. The extent to which anserine waterbirds may serve as reservoirs for enteric pathogens in the future and their limitations should be clarified. Comprehensive data are essential in reducing the risks of gastrointestinal eukaryote transmission from wild geese, ducks, and swans.

## Figures and Tables

**Table 1:** Waterfowl species and gastrointestinal micropathogens found in migrating Anatidea species worldwide from 1998 to 2023.

				Test Methods					
Pathogen/ Host Species	State	No. Tested	No. Positiv (%)	Microscopy	Epifluorescence	ELISA ^1^	Molecular Maker	Species/Genotype	Ref. ^2^
*Giardia*									
*Anas acuta*	USA	1	1 (100)	Not done	Fluorescent-antibody (FA) staining	Not done	Heat shock protein, giardin	*Giardia* sp.	[44]
*A. americana*	USA	3	2 (66)	Not done	Fluorescent-antibody (FA) staining	Not done	Heat shock protein, giardin	*Giardia* sp.	[44]
*A. c. carolinensis*	USA	6	0	Not done	Fluorescent-antibody (FA) staining	Not done	Heat shock protein, giardin	*Giardia* sp.	[44]
*A. disccors*	USA	4	1 (25)	Not done	Fluorescent-antibody (FA) staining	Not done	Heat shock protein, giardin	*Giardia* sp.	[44]
*A. platyrhynchos*	USA	51	13 (25.4)	Not done	Fluorescent-antibody (FA) staining	Not done	Heat shock protein, giardin	*Giardia* sp.	[44]
*A. platyrhynchos*	Poland	32	7 (22)	Wet smear, haematoxylin staining	FISH ^3^/MERIFLOR^TM^ *Cryptosporidium*/*Gardia* test kit	Not done	n.d.	*G. duodenalis*	[45]
*A. platyrhynchos*	Spain	4	2 (50)	Not done	AquaGlo^TH^ G/C Direct test	Not done	18S rRNA, ITS1-5-8S-ITS2	*G. duodenalis* assemblage A (A2), B, F	[43]
*Anser anser*	Poland	34	10 (29)	Wet smear, haematoxylin staining	FISH/MERIFLUOR^TM^ *Cryptosporidium*/*Gardia* test kit	Not done	Not done	*G. duodenalis*	[45]
*A. anser*	Iran	17	n.d. ^4^ (8.3)	Trichrome staining	Not done	Not done	Not done	*G. duodenalis*	[46]
*Aythaya fuligula*	Iran	16	n.d. (43.8)	Trichrome staining	Not done	Not done	Not done	*G. duodenalis*	[46]
*Branta canadensis*	USA	n.d.	n.d.	Not done	MERIFLUOR^TM^ *Cryptosporidium*/*Gardia* test kit; IFA ^5^	Not done	Beta-tubulin	*Giardia* sp.	[33]
*B. canadensis*	USA	234	0	Not done	AquaGlo^TH^ G/Comprehensive Kit	ProSpecT^® 6^ *Giardia* EZ Microplate Array	Not done	Not done	[47]
*B. c. maxima*	USA	18	3 (16.6)	Not done	Not done	monoclonal enzyme immunoassay (EIA)	Not done	Not done	[8]
*Cygnus olor*	Poland	33	4 (12)	Wet smear, haematoxylin staining	FISH/MERIFLUOR^TM^ *Cryptosporidium*/*Gardia* test kit	Not done	Not done	*G. duodenalis*	[45]
*Lophodytes cucullatus*	USA	1	0	Not done	Fluorescent-antibody (FA) staining	Not done	Heat shock protein, giardin	*Giardia* sp.	[44]
*Mergus merganser*	USA	3	1 (33)	Not done	Fluorescent-antibody (FA) staining	Not done	Heat shock protein, giardin	*Giardia* sp.	[44]
*M. merganser*	Poland	72	1 (1.5)	Wet smear, haematoxylin staining	FISH/MERIFLOR^TM^ *Cryptosporidium*/*Gardia* test kit	Not done	Not done	*G. duodenalis*	[45]
*Cryptosporidium*									
*Anas acuta*	USA	1	0	Not done	Fluorescent-antibody (FA) staining	Not done	18S rRNA	*Cryptosporidium* sp.	[44]
*A. americana*	USA	3	3 (100)	Not done	Fluorescent-antibody (FA) staining	Not done	18S rRNA	*Cryptosporidium* sp.	[44]
*A. c. carolinensis*	USA	6	3 (50)	Not done	Fluorescent-antibody (FA) staining	Not done	18S rRNA	*Cryptosporidium* sp.	[44]
*A. crecca*	Iran	36	n.d. (11.9)	Ziehl–Neelsen staining	Not done	Not done	Not done	*Cryptosporidium* sp.	[46]
*A. crecca*	Cyprus	20	0	Not done	Not done	Not done	18S rRNA		[48]
*A. disccors*	USA	4	2 (50)	Not done	Fluorescent-antibody FA) staining	Not done	18S rRNA	*Cryptosporidium* sp.	[44]
*A. penelope*	Iran	27	n.d. (11.2)	Ziehl–Neelsen staining	Not done	Not done	Not done	*Cryptosporidium* sp.	[46]
*A. platyrhynchos*	USA	51	23 (45)	Not done	Fluorescent-antibody (FA) staining	Not done	18S rRNA	*Cryptosporidium* sp.	[44]
*A. platyrhynchos*	Poland	200	13 (6.5)	Wet smear, Ziehl–Neelsen staining	IFA/MERIFLUOR^TM^ *Cryptosporidium*/*Gardia* test kit	ProSpecT^®^, *Cryptosporidium* Microplate Assay	Not done	*C. parvum*	[49]
*A. platyrhynchos*	Spain	4	2 (50)	Not done	AquaGlo^TH^ G/C Direct test	Not done	18S rRNA, hsp 70	*C. parvum* (genotype not determined)	[43]
*A. platyrhynchos*	New Zealand	80	1 (1.3)	Not done	Not done	Not done	18S rRNA	*Cryptosporidium* sp.	[50]
*A. platyrhynchos*	Iran	63	n.d. (22)	Ziehl–Neelsen staining	Not done	Not done	Not done	*Cryptosporidium* sp.	[46]
*A. platyrhynchos*	Algeria	31	1 (3.2)	Aniline-carbol-methyl violet staining	Not done	Not done	18S rRNA, actin, gp60	*C. baileyi*	[51]
*A. strepera*	Iran	34	n.d. (6.2)	Ziehl–Neelsen staining	Not done	Not done	Not done	*Cryptosporidium* sp.	[46]
*Anas* spp.	Cyprus	7	0	Not done	Not done	Not done	18S rRNA		[48]
*Anser anser*	Algeria	11	1 (9)	Aniline-carbol-methyl violet staining	Not done	Not done	18S rRNA, actin, gp60	*C. meleagridis*, IIIgA22G3R1	[51]
*A. anser*	UK	100	26 (26)	Not done	Not done	Not done	gp60	*C. parvum*; *C. andersoni* goose genotype	[32]
*A. fabalis*	Poland	192	5 (2.5)	Wet smear, Ziehl–Neelsen staining	IFA/MERIFLUOR^TM^ *Cryptosporidium*/*Gardia* test kit	ProSpecT^®^, *Cryptosporidium* Microplate Assay	Not done	*C. parvum*	[49]
*Branta canadensis*	USA	n.d.	n.d.		IFA/MERIFLUOR^TM^ *Cryptosporidium*/*Gardia* test kit		TRAP C2	*C. parvum* genotype 2	[33]
*B. canadensis*	USA	209	49 (23.4)	Not done	Not done	Not done	18S rRNA	*Cryptosporidium* goose genotype I, goose genotype II, *Cryptosporidium* duck genotype, *C. parvum*, *C. hominis*	[52]
*B. canadensis*	USA	161	11 (6.8)	Not done	Not done	Not done	18S rRNA	*Cryptosporidium* goose genotype	[53]
*B. canadensis*	USA	144	4 (2.8)	Phase contrast	Not done	ELISA	Not done	*Cryptosporidium* sp.	[19]
*B. canadensis*	New Zealand	80	4 (5)	Not done	Not done	Not done	18S rRNA	*Cryptosporidium* sp.	[50]
*B. c. maxima*	USA	18	14 (77)			Monoclonal enzyme immunoassay (EIA)			[8]
*Cygnus atratus*	New Zealand	80	2 (2.5)	Not done	Not done	Not done	18S rRNA	*Cryptosporidium* sp.	[50]
*C. cygnus*	Iran	44	n.d. (36)	Ziehl–Neelsen staining	Not done	Not done	Not done	*Cryptosporidium* sp.	[46]
*C. cygnus*	China	467	8 (1.7)	Not done	Not done	Not done	18S rRNA	*C. parvum*, *C. andersoni*, goose genotype II	[54]
*C. olor*	Poland	19	3 (15.8)	Wet smear, Ziehl-Neelsen staining	IFA/MERIFLUOR^TM^ *Cryptosporidium*/*Gardia* test kit	ProSpecT^®^, *Cryptosporidium* Microplate Assay	Not done	*C. parvum*	[49]
*C. olor*	Poland	33	4 (12.5)	Wet smear, Ziehl–Neelsen staining	FISH/MERIFLUOR^TM^ *Cryptosporidium*/*Gardia* test kit	Not done	Not done	*C. parvum*	[45]
*Lophodytes cucullatus*	USA	1	1 (100)	Not done	Fluorescent-antibody (FA) staining	Not done	18S rRNA	*Cryptosporidium* sp.	[44]
*Mergus merganser*	USA	3	2 (67)	Not done	Fluorescent-antibody (FA) staining	Not done	18S rRNA	*Cryptosporidium* sp.	[44]
*M. merganser*	Poland	72	2 (2.8)	Wet smear, Ziehl–Neelsen staining	IFA/MERIFLUOR^TM^ *Cryptosporidium*/*Gardia* test kit	ProSpecT^®^, *Cryptosporidium* Microplate Assay	Not done	*C. parvum*	[49]
*M. merganser*	USA	5	1 (20)	Phase contrast	Not done	ELISA	Not done	*Cryptosporidium* sp.	[19]
*Spatula clypeata*	Iran	23	n.d. (32.3)	Ziehl–Neelsen staining	Not done	Not done	Not done	*Cryptosporidium* sp.	[46]
*Tadoma ferruginea*	China	148	5 (3.4)	Ziehl–Neelsen staining	Not done	Not done	SSU rDNA sp70	*C. baileyi*	[55]
*Migratory duck* spp.	Japan	200	23 (11.5)	Not done	Not done	Not done	18S rRNA	*C. proventriculi*, *C. baileyi*	[56]
*Cyclospora*									
*Anas platyrhynchos*	Poland	200	0	Ziehl–Neelsen staining, sporulation test	Not done	Not done	18S rRNA	*Cyclospora* sp.	[49]
*Anser fabalis*	Poland	192	2 (0.5)	Ziehl–Neelsen staining, sporulation test	Not done	Not done	18S rRNA	*Cyclospora* sp.	[49]
*Cygnus olor*	Poland	19	1 (5.3)	Ziehl–Neelsen staining, sporulation test	Not done	Not done	18S rRNA	*Cyclospora* sp.	[49]
Microsporidia									
*Anas platyrhynchos*	Poland	28	5 (18)	Chromatrope-2R, calcofluor white M2R staining	FISH	Not done	Not done	*Encephalitozoon hellem*	[39]
*Anser anser*	Poland	34	3 (9)	Chromatrope-2R, calcofluor white M2R staining	FISH	Not done	Not done	*Encephalitozoon hellem*	[39]
*Cygnus cygnus*	China	467	35 (7.49)	Not done	Not done	Not done	IST ^7^	*Enterocytozoon bieneusi* (genotypes BEB6, EbpC, EbpA, Peru6, Nenan-III, Henan-IV, PtEb IX, CD9, CSW1, CSW 2, CSW 3)	[16]
*C. olor*	Poland	30	4 (13)	Chromatrope-2R, calcofluor white M2R staining	FISH	Not done	Not done	*Encephalitozoon hellem*	[39]

^1^ Enzyme-linked Immunosorbent Assay; ^2^ References; ^3^ Multiplexed fluorescence in situ hybridization combined with immunofluorescent antibody; ^4^ No data; ^5^ immunofluorescent antibody test; ^6^ *Cryptosporidium* microplate assay based on detection of *Cryptosporidium* specific antigens (CSA) in faecal specimens; ^7^ Internal transcribed spacer species.

## Data Availability

Not applicable.

## References

[1] WHO (2012). Research Priorities for Zoonoses and Marginalized Infections.

[2] Jones K.E., Patel N.G., Levy M.A., Storeygard A., Balk D., Gittleman J.L., Daszak P. (2008). Global trends in emerging infectious diseases. Nature.

[3] Fletcher S.M., Stark D., Harkness J., Ellis J. (2012). Enteric protozoa in the developed world: A public health perspective. Clin. Microbiol. Rev..

[4] Ryan U., Cacciò S.M. (2013). Zoonotic potential of *Giardia*. Int. J. Parasitol..

[5] Tsiodras S., Kelesidis T., Kelesidis I., Bauchinger U., Falagas M.E. (2008). Human infections associated with wild birds. J. Infect..

[6] Elmberg J., Berg C., Lerner H., Waldenström J., Hessel R. (2017). Potential disease transmission from wild geese and swans to livestock, poultry and humans: A review of the scientific literature from a One Health perspective. Infect. Ecol. Epidemiol..

[7] Hubálek Z. (2004). An annotated checklist of pathogenic microorganisms associated with migratory birds. J. Wildl. Dis..

[8] Kassa H., Harrington B., Bisesi M.S. (2001). Risk of Occupational Exposure to *Cryptosporidium, Giardia*, and *Campylobacter* Associated with the Feces of Giant Canada Geese. Appl. Occup. Environ. Hyg..

[9] Feng Y., Xiao L. (2011). Zoonotic potential and molecular epidemiology of *Giardia* species and giardiasis. Clin. Microbiol. Rev..

[10] Efstratiou A., Ongerth J.E., Karanis P. (2017). Waterborne transmission of protozoan parasites: Review of worldwide outbreaks—An update 2011–2016. Water Res..

[11] Zahedi A., Monis P., Deere D., Ryan U. (2021). Wastewater-based epidemiology-surveillance and early detection of waterborne pathogens with a focus on SARS-CoV-2, *Cryptosporidium* and *Giardia*. Parasitol. Res..

[12] Kucerova-Pospisilova Z., Carr D., Leitch G., Scanlon M., Visvesvara G.S. (1999). Environmental resistance of *Encephalitozoon* spores. J. Eukaryot. Microbiol..

[13] Jarroll E.L., Bingham A.K., Meyer E.A. (1981). Effect of chlorine on *Giardia lamblia* cyst viability. Appl. Environ. Microbiol..

[14] Hassan E.M., Örmeci B., DeRosa M.C., Dixon B.R., Sattar S.A., Iqbal A. (2021). A review of *Cryptosporidium* spp. and their detection in water. Water Sci. Technol..

[15] Totton S.C., O’Connor A.M., Naganathan T., Martinez B.A.F., Vriezen E.R., Torrence M.E., Sargeant J.M. (2021). A scoping review of the detection, epidemiology and control of *Cyclospora cayetanensis* with an emphasis on produce, water and soil. Epidemiol. Infect..

[16] Wang Y., Zhang K., Zhang Y., Wang K., Gazizova A., Wang L., Cao L., Zhang Y., Huang J., Cui Y. (2020). First detection of *Enterocytozoon bieneusi* in whooper swans (*Cygnus cygnus*) in China. Parasites Vectors.

[17] Ryan U., Zahedi A., Feng Y., Xiao L. (2021). An Update on Zoonotic *Cryptosporidium* Species and Genotypes in Humans. Animals.

[18] Weber R., Bryan R.T. (1994). Microsporidial infections in immunodeficient and immunocompetent patients. Clin. Infect. Dis..

[19] Ziegler P.E., Wade S.E., Schaaf S.L., Stern D.A., Nadareski C.A., Mohammed H.O. (2007). Prevalence of *Cryptosporidium* species in wildlife populations within a watershed landscape in southeastern New York State. Vet. Parasitol..

[20] Ruan Y., Xu X., He Q., Li L., Guo J., Bao J., Pan G., Li T., Zhou Z. (2021). The largest meta-analysis on the global prevalence of microsporidia in mammals, avian and water provides insights into the epidemic features of these ubiquitous pathogens. Parasites Vectors.

[21] Ortega Y.R., Sanchez R. (2010). Update on *Cyclospora cayetanensis*, a food-borne and waterborne parasite. Clin. Microbiol. Rev..

[22] Buret A.G., Cacciò S.M., Favennec L., Svärd S. (2020). Update on *Giardia*: Highlights from the seventh International *Giardia* and *Cryptosporidium* Conference. Parasite.

[23] Cinar H.N., Gopinath G., Murphy H.R., Almeria S., Duringan M., Choi D., Jang A.Y., Kim E., Kim R.Y., Choi S. (2020). Molecular typing of *Cyclospora cayetanensis* in produce and clinical samples using targeted enrichment of complete mitochondrial genomes and next-generation sequencing. Parasites Vectors.

[24] Reed K.D., Meece J.K., Henkel J.S., Shukla S.K. (2003). Birds, migration and emerging zoonoses: West nile virus, lyme disease, influenza A and enteropathogens. Clin. Med. Res..

[25] Zhang G.G., Chen L.X., Li S.H., Gao R.Y., Ru W.D., Liu D.P., Sun M.H., Hou Y.Q., Lu J. (2016). The current status of wintering population of whooper swan (*Cygnus cygnus*) at Sanmenxia reservoir region, China. Chin. J. Zool..

[26] Rushing C.S., Royle J.A., Ziolkowski D.J., Pardieck K.L. (2020). Migratory behavior and winter geography drive differential range shifts of eastern birds in response to recent climate change. Proc. Natl. Acad. Sci. USA.

[27] Aagaard K.J., Lonsdorf E.V., Thogmartin W.E. (2022). Effects of weather variation on waterfowl migration: Lessons from a continental-scale generalizable avian movement and energetics model. Ecol. Evol..

[28] Dieter R.A., Dieter R.S., Dieter R.A., Gulliver G. (2001). Zoonotic diseases: Health aspects of Canadian geese. Int. J. Circumpolar Health.

[29] Kassa H., Harrington B.J., Bisesi M.S. (2004). Cryptosporidiosis: A brief literature review and update regarding *Cryptosporidium* in feces of Canada geese (*Branta canadensis*). J. Environ. Health.

[30] Gorham T.J., Lee J. (2016). Pathogen Loading from Canada Geese Faeces in Freshwater: Potential Risks to Human Health Through Recreational Water Exposure. Zoonoses Public Health.

[31] Nagamori Y., Litherland M.A., Koons N.R., Linthicum A.R., Ramachandran A. (2022). Survey of zoonotic parasites and bacteria in faeces of Canada geese (*Branta canadensis*) in North-Central Oklahoma. Vet. Med. Sci..

[32] Wells B., Paton C., Bacchetti R., Shaw H., Stewart W., Plowman J., Katzer F., Innes E.A. (2019). *Cryptosporidium* Prevalence in Calves and Geese Co-Grazing on Four Livestock Farms Surrounding Two Reservoirs Supplying Public Water to Mainland Orkney, Scotland. Microorganisms.

[33] Graczyk T.K., Fayer R., Trout J.M., Lewis E.J., Farley C.A., Sulaiman I., Lal A.A. (1998). *Giardia* sp. cysts and infectious *Cryptosporidium parvum* oocysts in the feces of migratory Canada geese (*Branta canadensis*). Appl. Environ. Microbiol..

[34] Wang Y., Zhang K., Chen Y., Li X., Zhang L. (2021). *Cryptosporidium* and cryptosporidiosis in wild birds: A One Health perspective. Parasitol. Res..

[35] Malčeková B., Valenčáková A., Molnár L., Kočišová A. (2013). First detection and genotyping of human-associated microsporidia in wild waterfowl of Slovakia. Acta Parasitol..

[36] Graczyk T.K., Majewska A.C., Schwab K.J. (2008). The role of birds in dissemination of human waterborne enteropathogens. Trends Parasitol..

[37] Merkens M., Bradbeer D.R., Bishop C.A. (2012). Landscape and field characteristics affecting winter waterfowl grazing damage to agricultural perennial forage crops on the lower Fraser River delta, BC, Canada. Crop. Protect..

[38] Clark L., Matthews K.R., Sapers G.M., Gerba C.P. (2014). Disease risks posed by wild birds associated with agricultural landscapes. The Produce Contamination Problem.

[39] Slodkowicz-Kowalska A., Graczyk T.K., Tamang L., Jedrzejewski S., Nowosad A., Zduniak P., Solarczyk P., Girouard A.S., Majewska A.C. (2006). Microsporidian species known to infect humans are present in aquatic birds: Implications for transmission via water?. Appl. Environ. Microbiol..

[40] Widmer G., Carmena D., Kváč M., Chalmers R.M., Kissinger J.C., Xiao L., Sateriale A., Striepen B., Laurent F., Lacroix-Lamandé S. (2020). Update on *Cryptosporidium* spp.: Highlights from the Seventh International Giardia and Cryptosporidium Conference. Parasite.

[41] Cacciò S.M., Lalle M., Svärd S.G. (2018). Host specificity in the *Giardia duodenalis* species complex. Infect. Genet. Evol..

[42] Ryan U., Zahedi A. (2019). Molecular epidemiology of giardiasis from a veterinary perspective. Adv. Parasitol..

[43] Reboredo-Fernández A., Ares-Mazás E., Cacciò S.M., Gómez-Couso H. (2015). Occurrence of *Giardia* and *Cryptosporidium* in wild birds in Galicia (Northwest Spain). Parasitology.

[44] Kuhn R.C., Rock C.M., Oshima K.H. (2002). Occurrence of *Cryptosporidium* and *Giardia* in wild ducks along the Rio Grande River valley in southern New Mexico. Appl. Environ. Microbiol..

[45] Majewska A.C., Graczyk T.K., Słodkowicz-Kowalska A., Tamang L., Jedrzejewski S., Zduniak P., Solarczyk P., Nowosad A., Nowosad P. (2009). The role of free-ranging, captive, and domestic birds of Western Poland in environmental contamination with *Cryptosporidium parvum* oocysts and *Giardia lamblia* cysts. Parasitol. Res..

[46] Shemshadi B., Ranjbar-Bahadori S., Faghihzadeh-Gorji S. (2014). Occurrence of parasitic protozoa in wild waterfowl in southern coastal Caspian sea lagoon. Iran J. Vet. Med..

[47] Ayers C.R., DePerno C.S., Moorman C.E., Stibbs H.H., Faust A.M. (2014). Survey of Canada goose feces for presence of *Giardia*. Hum. Wildl. Interact..

[48] Hasapis K.A., Charalambidou I., Tsouma E., Sotiriadi K., Kassinis N., Schou C., Karanis P. (2023). First detection of *Cryptosporidium proventriculi* from wild birds in Cyprus. Parasitol. Res..

[49] Majewska A.C., Kosiński Z., Werner A., Sulima P., Nowosad P. (2001). Pasożytnicze Pierwotniaki Jelitowe: Nowe Wodnopochodne Zagrożenie Zdrowia Publicznego.

[50] Leśniańska K., Perec-Matysiak A. (2017). Wildlife as an environmental reservoir of *Enterocytozoon bieneusi* (Microsporidia)—Analyses of data based on molecular methods. Ann. Parasitol..

[51] Elkarim Laatamna A., Holubova N., Sak B., Kvac M. (2017). *Cryptosporidium meleagridis* and *C. baileyi* (Apicomplexa) in domestic and wild birds in Algeria. Folia Parasitol..

[52] Zhou L., Kassa H., Tischler M.L., Xiao L. (2004). Host-adapted *Cryptosporidium* spp. in Canada geese (*Branta canadensis*). Appl. Environ. Microbiol..

[53] Jellison K.L., Distel D.L., Hemond H.F., Schauer D.B. (2004). Phylogenetic analysis of the hypervariable region of the 18S rRNA gene of *Cryptosporidium* oocysts in feces of Canada geese (*Branta canadensis*): Evidence for five novel genotypes. Appl. Environ. Microbiol..

[54] Wang K., Gazizova A., Wang Y., Zhang K., Zhang Y., Chang Y., Cui Y., Zhang Y., Zhang S., Zhang L. (2019). First Detection of *Cryptosporidium* spp. in Migratory Whooper Swans (*Cygnus cygnus*) in China. Microorganisms.

[55] Amer S., Wang C., He H. (2010). First detection of *Cryptosporidium baileyi* in Ruddy Shelduck (*Tadorna ferruginea*) in China. J. Vet. Med. Sci..

[56] Salama R.Y., Abdelbaset A.E., Takeda Y., Imai K., Ogawa H., Igarashi M. (2020). Molecular characterization of *Cryptosporidium* spp. from migratory ducks around Tokachi subprefecture, Hokkaido, Japan. J. Vet. Med. Sci..

[57] Dong S., Yang Y., Wang Y., Yang D., Yang Y., Shi Y., Li C., Li L., Chen Y., Jiang Q. (2020). Prevalence of *Cryptosporidium* Infection in the Global Population: A Systematic Review and Meta-analysis. Acta Parasitol..

[58] Holubová N., Tůmová L., Sak B., Hejzlarová A., Konečný R., McEvoy J., Kváč M. (2020). Description of *Cryptosporidium ornithophilus* n. sp. (Apicomplexa: Cryptosporidiidae) in farmed ostriches. Parasites Vectors.

[59] Ryan U.M., Feng Y., Fayer R., Xiao L. (2021). Taxonomy and molecular epidemiology of *Cryptosporidium* and *Giardia*—A 50 year perspective (1971-2021). Int. J. Parasitol..

[60] Ortega Y.R., Gilman R.H., Sterling C.R. (1994). A new coccidian parasite (Apicomplexa: Eimeriidae) from humans. J. Parasitol..

[61] Hofstetter J.N., Nascimento F.S., Park S., Casillas S., Herwaldt B.L., Arrowood M.J., Qvarnstrom Y. (2019). Evaluation of Multilocus Sequence Typing of *Cyclospora cayetanensis* based on microsatellite markers. Parasite.

[62] Solarczyk P. (2021). Host Range of *Cyclospora* Species: Zoonotic Implication. Acta Protozool..

[63] Almeria S., Cinar H.N., Dubey J.P. (2019). *Cyclospora cayetanensis* and Cyclosporiasis: An Update. Microorganisms.

[64] Relman D.A., Schmidt T.M., Gajadhar A., Sogin M., Cross J., Yoder K., Sethabutr O., Echeverria P. (1996). Molecular phylogenetic analysis of *Cyclospora*, the human intestinal pathogen, suggests that it is closely related to *Eimeria* species. J. Infect. Dis..

[65] Han B., Pan G., Weiss L.M. (2021). Microsporidiosis in Humans. Clin. Microbiol. Rev..

[66] Anane S., Attouchi H. (2010). Microsporidiosis: Epidemiology, clinical data and therapy. Gastroenterol. Clin. Biol..

